# Epidemiology and Etiology of Acute Pancreatitis in Urban and Suburban Areas in Shanghai: A Retrospective Study

**DOI:** 10.1155/2018/1420590

**Published:** 2018-08-12

**Authors:** Junjie Fan, Ling Ding, Yingying Lu, Junyuan Zheng, Yue Zeng, Chunlan Huang

**Affiliations:** Department of Gastroenterology, Shanghai General Hospital, Shanghai Jiao Tong University, Shanghai 200080, China

## Abstract

**Aim:**

To investigate the epidemiology, etiology, and severity of acute pancreatitis (AP) in urban and suburban areas of Shanghai in 2011 and 2016.

**Methods:**

A retrospective study of patients admitted to Shanghai General Hospital (urban and suburban campuses) with AP in 2011 and 2016 was undertaken. Patients were divided into acute biliary pancreatitis (ABP), hypertriglyceridemic pancreatitis (HTGP), alcoholic pancreatitis, and pancreatitis of other causes according to etiology. Severity of AP was divided into mild AP (MAP), moderately severe AP (MSAP), and severe AP (SAP).

**Results:**

AP patients in the suburban area increased more rapidly than those in the urban area. The mean onset age of AP in the urban area in 2016 was older than that in the suburban area (*p* < 0.05). The suburban patients in 2016 have significantly younger mean onset age than those in 2011 (*p* < 0.05). HTGP incidence in suburban patients increased from 2011 to 2016, which changed little in the urban area. Urban females were more likely to develop HTGP than suburban ones in 2011, which reversed in 2016. As to the male patients, the incidence of HTGP increased in both urban and suburban areas. Nonelderly (<60 years old) patients had higher HTGP incidence than elderly ones in both 2011 and 2016. The descending trend of SAP in the suburban area was more obvious than that in the urban area. The length of hospitalization decreased from 2011 to 2016, especially in SAP patients.

**Conclusions:**

AP patients increased more rapidly in the suburban area of Shanghai with younger onset age. The incidence of HTGP increased significantly in the suburban area, reminding of the prevention and screening of HTG.

## 1. Introduction

The incidence of acute pancreatitis (AP) has increased over the past few decades in much of the world [1, 2], although there are a few reports differing from the increasing trend [3]. AP has become the most common inpatient gastrointestinal diagnosis and costs an estimated 2.6 billion dollars per year in the United States [4], which is still increasing [5].

Worldwide, the cause of AP varies across countries and regions. A study across Europe has found that gallstone is the dominant etiology in Southern Europe and alcohol in Eastern Europe with intermediate ratios in Northern and Western Europe. Research investigating the socioeconomic patterning of harmful alcohol consumption has also found that lower socioeconomic status (SES) groups drink more heavily [6], consistent with the research that alcoholic AP is linked strongly with social deprivation [7].

Knowing the pattern of illness including etiology and severity in a given population is important for planning the prevention and management for AP. Shanghai is a mega city in Eastern China with a population of more than 20 million, up to 42% of whom are floating migrants not residing in their places of household registration [8]. Most of the floating population reside in the suburban area of Shanghai due to the cheaper living and rented accommodation costs. Till now, the etiology and epidemiology of AP in urban and suburban areas of Shanghai have not been reported.

Shanghai General Hospital is one the largest hospitals in Shanghai with two campuses, the north Hongkou campus and the south Songjiang campus. The northern campus is located in the urban area of Shanghai while the southern one in the suburban area. Most of the patients committed into the northern campus are local residents in the urban area, while most of those in the southern campus are local residents in the suburban area and floating migrants. We retrospectively analyzed the histories and clinical data of patients with AP committed to the two campuses. Our aim is to assess the etiology, severity, and epidemiology of AP in urban and suburban areas of Shanghai.

## 2. Methods

We followed the methods of Huang et al. [9]. Our retrospective study included patients that were diagnosed with AP admitted to Shanghai General Hospital in China in 2011 and 2016. All human studies were approved by the Ethics Committee of Shanghai General Hospital and were performed in accordance with ethical standards. A diagnosis of AP was confirmed based on the revised Atlanta classification [10], which also presents the severity classification of AP. The patients who did not meet the criteria of AP or were less than 16 years old were excluded from the study. Two independent investigators using a standard data collection instrument confirmed the patient age, etiology, incidence of systematic and local complications, and outcome.

Transabdominal ultrasonography was performed to diagnose acute biliary pancreatitis (ABP). An alanine aminotransferase (ALT) level of >150 U/L within 48 hours after the onset of symptoms was used to confirm ABP, with a positive predictive value > 85% [11]. According to the Chinese guideline for acute pancreatitis and study of hypertriglyceridemic AP (HTGP), patients with AP that had a triglyceride (TG) level of ≥11.3 mmol/L (1000 mg/dL) or between 5.65 and 11.3 mmol/L (500 and 1000 mg/dL) but with lipemic serum were diagnosed with HTGP based on the absence of biliary disease, alcohol, or medication abuse [12, 13]. Alcoholic AP was defined with the history of alcohol abuse or alcoholic binge prior to the episode of AP and without other evidence of AP cause from the history [14]. Besides ABP, HTGP, and alcoholic AP, all the remaining ones fell into the category of AP with other etiologies (other AP) in our research. Other AP was diagnosed with miscellaneous causes including pancreatic cancer, medications, trauma, hyperparathyroidism, recent invasive procedure, and idiopathic AP as well.

Acute pancreatitis was classified according to three degrees of severity in the revised Atlanta classification: mild AP (MAP), moderately severe AP (MSAP), and severe AP (SAP) [10]. MAP is characterized by the absence of organ failure and local (or systemic) complications, MSAP was characterized by the presence of transient organ failure or local (or systemic) complications, and SAP was characterized by persistent organ failure. Transient organ failure was defined as organ failure with a duration of <48 hours whereas persistent organ failure was defined as organ failure that was not resolved after 48 hours of treatment [10]. Systematic complications included organ failure, systemic inflammatory response syndrome (SIRS), and death. Organ failure is defined as a score of 2 or more for one of the three organ systems (respiratory, cardiovascular, and renal) using the modified Marshall scoring system [10].

The AP patients were divided into nonelderly (<60 years old) groups and elderly (≥60 years old) ones according to the age.

Statistical analysis was performed using SPSS 19.0. The age and length of hospitalization were expressed as the mean ± standard deviation. Differences in mean scores were compared using Student's *t*-test. *p* values of <0.05 were considered statistically significant. Patient demographic and clinical characteristics have been reported as percentages for discrete characteristics.

## 3. Results

### 3.1. General Information

**Figure 1:**
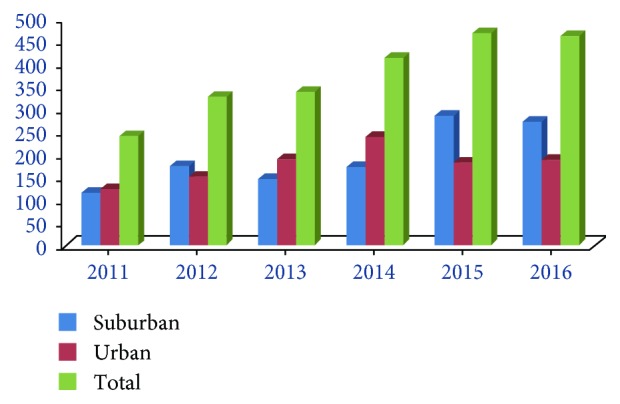
Number of AP patients. Patients with AP admitted at Shanghai General Hospital in China and the trend by year from 2011 to 2016. The retrospective study included 694 patients with AP, including 125 AP patients in the urban area and 116 in the suburb in 2011 and 179 in the urban area and 274 in the suburb in 2016.

**Figure 2:**
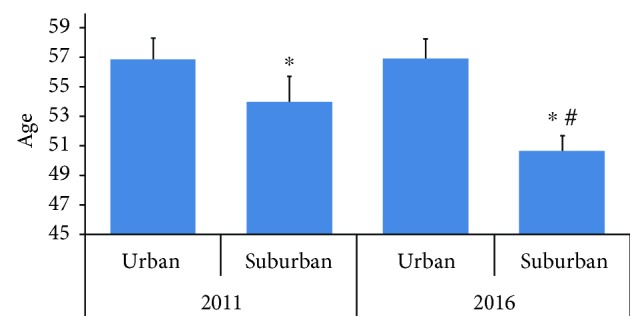
The mean ages of AP onset in urban and suburban areas. The mean ages of AP onset in the urban and suburban areas showed a significant difference in 2011 (56.858 ± 1.487 versus 54.009 ± 2, resp., *p* > 0.05), whereas in 2016, the mean age in the urban area was significantly higher than that in the suburb (56.917 ± 1.376 versus 50.665 ± 1.032, resp., *p* < 0.05). ^∗^*p* < 0.05 versus corresponding Urban group and ^#^*p* < 0.05 versus corresponding group in 2011.

A total of 694 patients met the criteria for AP inclusion in the study. The AP patients in urban and suburban areas increased from 125 and 116 in 2011 to 179 and 274 in 2016, respectively. The total number of AP patients increased annually in both areas, and the growth rate in the suburban area was higher than that in the urban area (Figure 1). The mean ages of AP onset in urban and suburban areas showed no significant difference in 2011 (56.858 ± 1.487 versus 54.009 ± 2, resp., *p* > 0.05), whereas in 2016, the mean age in the urban area was significantly higher than that in the suburban area (56.917 ± 1.376 versus 50.665 ± 1.032, resp., *p* < 0.05) (Figure 2).

### 3.2. Different Etiologies of Acute Pancreatitis

**Figure 3:**
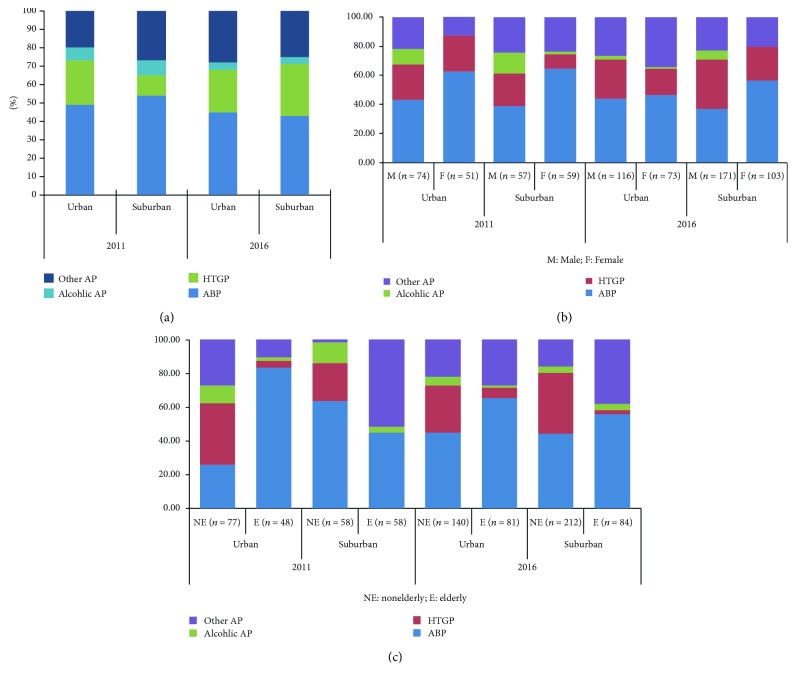
Etiology of acute pancreatitis. The etiology of AP patients including ABP, alcoholic AP, HTGP, and other AP according to urban and suburban areas in 2011 and 2016. (a) The incidence of HTGP in the suburban area was much less than that in the urban area in 2011 (11% versus 24%, resp.), which reversed in 2016 (28% versus 23%, resp.). (b) Etiologies according to sex distribution. The ABP was higher in female patients than in male patients no matter whether in the urban area or in the suburban area both in 2011 and in 2016. (c) Etiologies according to age distribution. ABP and HTGP were the two main causes in nonelderly patients, while in elderly ones, the only main cause was ABP with very little incidence of HTGP.

The distribution of AP etiology, including ABP, alcoholic AP, HTGP, and other AP cases, was analyzed according to urban and suburban areas in 2011 and 2016. Biliary etiology was the most frequent primary cause of AP in both areas in 2011 (49% versus 54%, resp.) and in 2016 (45% versus 43%, resp.). In 2011, the proportion of alcoholic AP was almost the same in both urban and suburban areas (7% versus 8%, resp.), both of which fell to nearly 4% in 2016. HTGP in the suburban area increased at a higher rate than that in the urban area. The incidence of HTGP in the suburban area was much lower than that in the urban area in 2011 (11% versus 24%, resp.), which reversed in 2016 (28% versus 23%, resp.) (Figure 3(a)).

### 3.3. Etiologies according to Sex and Age Distribution

The ABP incidence was higher in female patients in both areas. In 2011, the percentage of ABP of female patients in the urban and suburban areas was much higher than that of male patients (75% versus 43% and 64% versus 39%, resp.), which fell to 47% versus 44% and 56% versus 37%, respectively, in 2016. The alcoholic AP was lower in female patients than in male patients both in the urban and suburban areas in 2011 (0% versus 11% and 2% versus 14%, resp.) and 2016 (1% versus 3% and 0% versus 6%, resp.). The descending trend of alcoholic AP was showed in male patients in the urban and suburban areas from 2011 to 2016 (11% versus 14% and 3% versus 6%, resp.). HTGP incidence in two areas varied according to the sex distribution. In 2011, the percentage of HTGP of female patients in the urban area was higher than that in the suburban area (24% versus 10%, resp.), which reversed in 2016 (18% versus 23%, resp.). As to the male patients, the incidence of HTGP increased both in the urban and suburban areas. The HTGP incidence in male patients in the urban area increased from 24% in 2011 to 27% in 2016, while that in the suburban area from 23% to 34%. In 2011, the percentage of HTGP male patients was much higher than that of female patients in the suburban area (23% versus 10%, resp.), while little difference was showed in the urban area (24% versus 29%, resp.). In 2016, the percentage of HTGP male patients was higher than that of female patients in the urban and suburban areas (34% versus 23% and 27% versus 18%, resp.) (Figure 3(b)).

The influence of age on the etiologies of AP patients was also studied. ABP and HTGP were two main causes in nonelderly patients, while in elderly ones, the only main cause was ABP and with very little incidence of HTGP. As to the nonelderly patients, the HTGP incidence in the urban area decreased from 36% in 2011 to 28% in 2016, while that in the suburban area increased from 22% to 36% (Figure 3(c)).

### 3.4. Severity of Acute Pancreatitis

**Figure 4:**
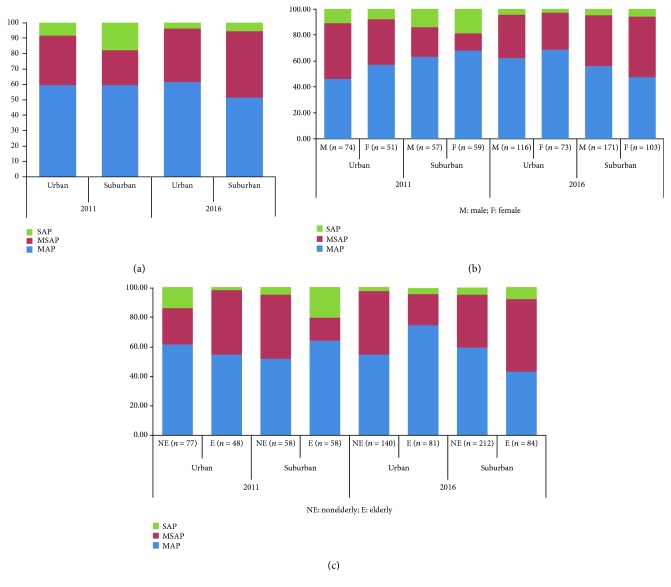
Severity of acute pancreatitis. The severity of AP patients according to urban and suburban areas in 2011 and 2016. (a) The proportion of SAP in the suburban area (18%) was higher than that in the urban area (8%) in 2011. (b) Severity according to sex distribution. According to the sex distribution, the proportion of SAP was higher in females in the suburban area (19% versus 14%, resp., in 2011 and 6% versus 3%, resp., in 2016), while the proportion was higher in males in the urban area (14% versus 10%, resp., in 2011 and 5% versus 4%, resp., in 2016). (c) Severity according to age distribution.

The severity of AP in the two areas was studied. The percentage of SAP in the suburban area (18%) was higher than that in the urban area (8%) in 2011 but more similar in 2016 (6% versus 4%, resp.). The SAP incidence decreased in both areas, and the descending trend in the suburban area was more obvious (18% versus 6%, resp.) than that in the urban area (8% versus 4%, resp.) (Figure 4(a)).

### 3.5. Severity of Acute Pancreatitis according to Etiology Distribution

The severity of AP varied according to the etiologies, which also changed over time. HTGP has become the most common cause of SAP, and the percentage of HTGP in SAP patients increased from 26.7% in 2011 into 50.0% in 2016. No alcoholic pancreatitis was found in SAP in 2016, which was only 6.7% in 2011. The ABP in SAP changed a little from 30% in 2011 to 31.8% in 2016 (Table 1).

### 3.6. Severity of Acute Pancreatitis according to Sex and Age Distribution

According to the sex distribution, the proportion of SAP was higher in females in the suburban area (19% versus 14%, resp., in 2011 and 6% versus 3%, resp., in 2016), while the proportion was higher in males in the urban area (14% versus 10%, resp., in 2011 and 5% versus 4%, resp., in 2016) (Figure 4(b)).

There was no obvious difference between the two areas according to age distribution. In 2011, in the urban area, the SAP percentage was higher in the nonelderly (14%) than in the elderly (2%), while reversely in the suburban area, SAP was higher in the elderly (21%) than in the nonelderly (5%). In 2016, the SAP percentage in the two areas in the elderly and nonelderly ranged between 2% and 8% (Figure 4(c)).

### 3.7. Stay of Hospitalization according to Severity

**Figure 5:**
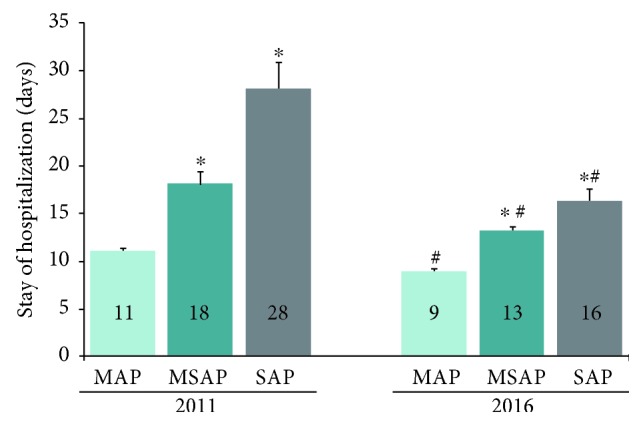
Hospitalization stay according to severity. It was found that the length of hospitalization (LOH) of AP decreased significantly over time in all the MAP, MSAP, and SAP groups. The LOH of SAP in 2016 was significantly shorter than that in 2011 (16 ± 1.35 days versus 28 ± 2.85 days, resp., *p* < 0.05). ^∗^*p* < 0.05 versus corresponding MAP group; ^#^*p* < 0.05 versus corresponding group in 2011.

It was found that the length of hospitalization (LOH) of AP decreased significantly over time in all the MAP, MSAP, and SAP groups. The LOH of SAP in 2016 was significantly shorter than that in 2011 (16 ± 1.35 days versus 28 ± 2.85 days, resp.) (Figure 5).

## 4. Discussions

This is the first study investigating the epidemiology, etiology, and severity of AP in urban and suburban areas in Shanghai. AP patients from two campuses of the same large hospital located in two areas were focused on as the model to fulfill the study.

In our study, AP hospitalization increased annually both in urban and suburban areas, and the increasing rate in the suburban area was much higher. A previous study showed that the peak incidence of pancreatitis for both men and women was between 40 and 70 years of age, with a male predominance in all age groups [15]. We found that the AP patients in the suburban area were associated with younger mean age than those in the urban area. A comparison of the mean age of onset between 2011 and 2016 showed little change in the urban area and significant reduction in the suburban area. According to data published by the Bureau of Statistics of Shanghai, up to 31.6% of the total citizens in Shanghai were over 60 in 2016, 5.0% higher than those in 2015. Nowadays, the aggravating trend of aging population brings impact to the social health system in Shanghai. The reducing trend of age onset in the suburban area might be attributed to the huge floating population in the working age.

Gallstones and excess alcohol consumption are the leading causes of AP in the developed countries, followed by the hypertriglyceridemia (HTG) as the third most common cause of AP [16]. In our present study, however, although ABP was still the most frequent etiology of pancreatitis, HTGP was ranked as the second most common etiology both in the urban and suburban areas. The ABP-to-HTGP ratio was nearly 2 : 1. The prevalence of HTGP in both areas in 2011 and 2016 was much higher than what had been previously reported as 9% [17].

The risk of AP in patients with serum triglycerides > 1000 and >2000 mg/dL is ~5% and 10% to 20%, respectively [18]. The prevalence of HTG is high in developed countries such as the United States and Russia but with rare severe ones [19, 20]. A common cause of HTG is seen in conditions with insulin resistance, such as metabolic syndrome (MetS) and type 2 diabetes mellitus. An increase in TG production under these conditions may be due to excess free fatty acids returning to the liver, as well as an increased de novo TG production [21]. MetS is defined as a cluster of metabolic abnormalities including central obesity, hypertension, high plasma triglycerides, decreased high-density lipoprotein (HDL) cholesterol, and glucose intolerance. Having any three of the five abnormal indicators above has been defined as MetS [22]. A previous study showed the unstandardized prevalence of MetS elevated from 29.65% (2005) to 45.49% (2014) from a community study in China. The higher incidence of MetS was mainly attributed to more fasting blood glucose (FBG) and TG abnormality but not waist circumference (WC), blood pressure (BP) and HDL, and excess sugar and lipid-rich food intake (classic western food); underurbanization may be the underlying cause [23]. The same group also showed a distinct age-related prevalence of MetS between genders in a dramatically changed East China including Shanghai and 7 other provinces, in which the TG disorders played an important role [24]. HTG also frequently coexists with other secondary conditions, including poor diet, alcohol use, and obesity [25]. Weight reduction, regular physical exercise, and restriction of alcohol intake reduce plasma TG levels [26]. Although no definite TG survey in the floating population was available, it has been showed that tobacco use, excessive alcohol use, insufficient intake of vegetable and fruit, physical inactivity, and overweight or obesity were high in the floating population in China [27]. All these might partly explain why the prevalence of AP induced by HTG increased dramatically in recent years in the suburban area, especially in nonelderly patients in our research.

It should be recommended to disseminate health information and ensure health check-ups, especially in the rapid urbanization areas and in the floating population. When the TG concentration is very high (>500 mg/dL and especially if >1000 mg/dL), the primary goal of therapy is to reduce the triglyceride level to <500 mg/dL for the intent of reducing the risk of pancreatitis [28, 29].

The proportion of alcoholic AP was lower than 10% in both the urban and suburban areas, which was much lower in 2016 and was inconsistent with the researches in the western countries. The alcohol was the second common etiology according to several researches, and the proportion of alcoholic AP varied from 22% to 41% [7, 30, 31]. The alcoholic AP was reported to be linked with high levels of alcohol consumption and social deprivation [7]. The proportion of alcoholic AP in the suburban area was not as high as we had expected. Alcohol consumption has remained steady or has even decreased in most countries during recent decades. Heavy alcohol consumption is known to be tightly associated with pancreatitis; however, the relationship between less alcohol intake and pancreatitis episode has not been well defined. It was even reported that moderate alcohol intake is protective against all types of pancreatitis in women and against recurrent acute (RAP)/chronic pancreatitis (CP) in men [32].

Among the cases of biliary, alcoholic, and HTG etiologies, 30.0%, 6.7%, and 26.7%, respectively, were SAP in 2011, while 31.8%, 0%, and 50.0%, respectively, were SAP in 2016. It was found that the severity of AP according to the etiologies changed over time, and HTG has become the most common cause of SAP in 2016. It might partly be attributed to the increasing incidence of HTGP. To date, it is still controversial whether HTG causes more severe AP than other etiologies [17]. On the other hand, although the exact role of HTG in AP has not been fully elucidated [18], the most widely accepted theory proposes that excess triglycerides are hydrolyzed by pancreatic lipase forming high concentrations of cell-damaging free fatty acids (FFA) [17].

It was found that the length of hospitalization (LOH) of AP decreased significantly over time in all the MAP, MSAP, and SAP groups in our study. A few prior studies have shown that the presence of comorbidities, longer duration of fasting period, oral refeeding intolerance, need for abdominal cross-sectional imaging, endoscopic retrograde cholangiopancreatography (ERCP) during hospitalization, and low volume of fluid resuscitation within 24 h of presentation are related to increased LOH in mild AP patients [33]. As to the SAP patients, prompt recognition of organ dysfunction during the AP course is the important step. Improved ICU care, early enteral nutrition support, primarily conservative treatment strategy, and a revolved minimally invasive approach have probably contributed to a better outcome and shortened hospitalization period [11, 34]. Therapeutic plasma exchange can significantly decrease TG levels and reduce inflammatory cytokines in the early stage of HTGP [35] to prevent the HTG from accelerating the AP course [18].

A limitation of this study is that the research was held in one hospital which might lead to bias of the analysis. Further research based on multiple centers might give out more clear insights into the epidemiology of AP in urban and suburban areas in Shanghai.

## 5. Conclusions

AP patients increased more rapidly in the suburban area of Shanghai with younger onset age, partly because of the high proportion of floating people. The proportion of HTGP increased significantly in the suburban area, reminding of the prevention and screening of HTG.

## Figures and Tables

**Table 1:** Severity of acute pancreatitis according to etiologies distribution.

	2011			2016		
	MAP	MSAP	SAP	MAP	MSAP	SAP
ABP (*n*, %)	79 (55.6)	33 (9.3)	14 (30.0)	110 (42.8)	84 (45.7)	7 (31.8)
HTGP (*n*, %)	23 (16.2)	13 (19.4)	7 (26.7)	62 (24.1)	49 (26.6)	11 (50.0)
Alcoholic pancreatitis (*n*, %)	11 (7.7)	4 (6.0)	3 (6.7)	16 (6.2)	3 (1.6)	0 (0.0)
Other AP (*n*, %)	29 (20.4)	17 (25.4)	11 (36.7)	69 (26.8)	48 (26.1)	4 (18.2)

Severity of acute pancreatitis according to etiology distribution. The HTGP has become the most common cause of SAP, and the proportion of HTGP in SAP patients increased from 26.7% in 2011 to 50.0% in 2016.

## Data Availability

The data used to support the findings of this study are available from the corresponding author upon request.
